# An Artificial Neural Network-Based Algorithm for Evaluation of Fatigue Crack Propagation Considering Nonlinear Damage Accumulation

**DOI:** 10.3390/ma9060483

**Published:** 2016-06-17

**Authors:** Wei Zhang, Zhangmin Bao, Shan Jiang, Jingjing He

**Affiliations:** School of Reliability and Systems Engineering, Beihang University, Haidian District, Beijing 100089, China; baozhangmin@icloud.com (Z.B.); jshan.susan@gmail.com (S.J.); 09722@buaa.edu.cn (J.H.)

**Keywords:** fatigue crack growth, artificial neural network, nonlinear multivariable function, retardation, loading interaction

## Abstract

In the aerospace and aviation sectors, the damage tolerance concept has been applied widely so that the modeling analysis of fatigue crack growth has become more and more significant. Since the process of crack propagation is highly nonlinear and determined by many factors, such as applied stress, plastic zone in the crack tip, length of the crack, *etc.*, it is difficult to build up a general and flexible explicit function to accurately quantify this complicated relationship. Fortunately, the artificial neural network (ANN) is considered a powerful tool for establishing the nonlinear multivariate projection which shows potential in handling the fatigue crack problem. In this paper, a novel fatigue crack calculation algorithm based on a radial basis function (RBF)-ANN is proposed to study this relationship from the experimental data. In addition, a parameter called the equivalent stress intensity factor is also employed as training data to account for loading interaction effects. The testing data is then placed under constant amplitude loading with different stress ratios or overloads used for model validation. Moreover, the Forman and Wheeler equations are also adopted to compare with our proposed algorithm. The current investigation shows that the ANN-based approach can deliver a better agreement with the experimental data than the other two models, which supports that the RBF-ANN has nontrivial advantages in handling the fatigue crack growth problem. Furthermore, it implies that the proposed algorithm is possibly a sophisticated and promising method to compute fatigue crack growth in terms of loading interaction effects.

## 1. Introduction

As the damage tolerance concept is now widely accepted and applied in the aerospace and aviation industries, it has become increasingly important to analyze how a fatigue crack grows. Linear elastic fracture mechanics (LEFM), moreover, is the fundamental theory for establishing the analytical model of fatigue crack propagation. Paris and Erdogan [1] correlate the stress intensity factor (SIF) range with the fatigue crack growth rate, and propose this seminal model as Equation (1).

(1) $$\frac{da}{dN}=C_{P}{\left(\Delta K\right)}^{m_{P}}$$

where Δ*K* is the SIF range and *C_P_* and *m_P_* are the fitting parameters. This equation shows a linear relationship between *da*/*dN* and Δ*K* in the log-log coordinate. However, a major limitation of the Paris equation is that *C_P_* has to change along with the variation of the stress ratio (*R*). Additionally, it is only applicable to the linear region without the consideration of the threshold SIF (Δ*K_th_*) and the critical SIF (*K_c_*).

To perfect the Paris equation, many researchers have attempted to make modifications in order to involve more nonlinear factors. Forman *et al.* [2] take the effects of *R* and *K_c_* into consideration and propose a modified model as shown in Equation (2).

(2) $$\frac{da}{dN}=\frac{C_{F}{\left(\Delta K\right)}^{m_{F}}}{\left(1-R\right)K_{c}-\Delta K}$$

where *C_F_* and *m_F_* are the fitting parameters. Furthermore, based on the Paris equation, some researchers develop more general formulas by employing additional parameters to account for nonlinearity, such as the NASGRO formula [3] in Equation (3):

(3) $$\frac{da}{dN}=C_{Na}{\left[\frac{\left(1-f\right)}{\left(1-R\right)}\Delta K\right]}^{n}\frac{{\left(1-\frac{\Delta K_{th}}{\Delta K}\right)}^{P_{Na}}}{{\left(1-\frac{K_{\text{max}}}{K_{c}}\right)}^{q_{Na}}}$$

where *C_Na_*, *P_Na_* and *q_Na_* are the fitting parameters; and *f* is Newman’s [4] crack opening function determined by the experimental measurement.

The series of models discussed above are put forward to illustrate the nonlinear relationship between the crack growth rate and the SIF range for constant amplitude loadings. Moreover, many researchers have investigated the loading interaction effect, which is complicated and of great significance to the variable amplitude loading. In this paper, the constant amplitude loading with overload, which is the typical and simple variable amplitude loading, is studied. Wheeler proposes a plastic-zone-based model [5] to describe the crack growth retardation caused by overload as shown in Equation (4).

(4) $$\{\begin{array}{c} {\left(\frac{da}{dN}\right)}_{VA}=\gamma \frac{da}{dN}\\ \gamma ={\left(\frac{r_{p,i}}{\lambda }\right)}^{m} \end{array}$$

where *γ* is the retardation factor; *r_p,i_* is the size of current plastic zone; *λ* is the distance between the current crack tip and the edge of the plastic zone caused by overload; and m is the fitting parameter. De Koning [6] also develops a plastic-deformation-based model to deal with the overload effect. Wheeler’s and Koning’s models support that the plastic zone (monotonic plastic zone or reversed plastic zone) is the key parameter to correlate with loading interaction effects in the fatigue crack growth calculation.

Most studies focus on accurately quantifying the nonlinear relationship between the crack growth rate and the driving parameters by using an explicit and simple function. To achieve these goals, many studies have been undertaken to introduce more parameters to construct a formula which can fit the experimental data better. However, the current formulas are not flexible enough to positively handle all the situations.

Overall, the process of fatigue crack growth is a nonlinear and multivariable problem under both constant and variable amplitude loading. Fortunately, the artificial neural network (ANN) has an excellent ability to fit the nonlinear multivariable relationship, which makes it a sophisticated and promising approach to the fatigue crack growth problem. ANN is a family of algorithms based on the imitation of biological neural networks. It has the strong ability to estimate the tendency of nonlinear and multivariable functions based on a large amount of data [7]. Thanks to these advantages, ANN is widely applied to damage estimation in the material sciences [8,9,10]. Furthermore, it is used to deal with some fracture problems including creep, fatigue and even corrosion fatigue [11,12,13,14,15].

A novel ANN-based algorithm is proposed in this paper to evaluate the process of fatigue crack growth. In the following sections, the ANN is first established and its training outlined. While establishing the ANN, the equivalent SIF is used to account for the influence of the loading history. Subsequently, the ANN-centered algorithm is developed and validated by using experimental data under the constant amplitude loading with different stress ratios or overloads. Some classical models are also employed for comparison. In the final section, some conclusions and considerations are given.

## 2. Methodology

### 2.1. Radial Basis Function Artificial Neural Network

**Figure 1:**
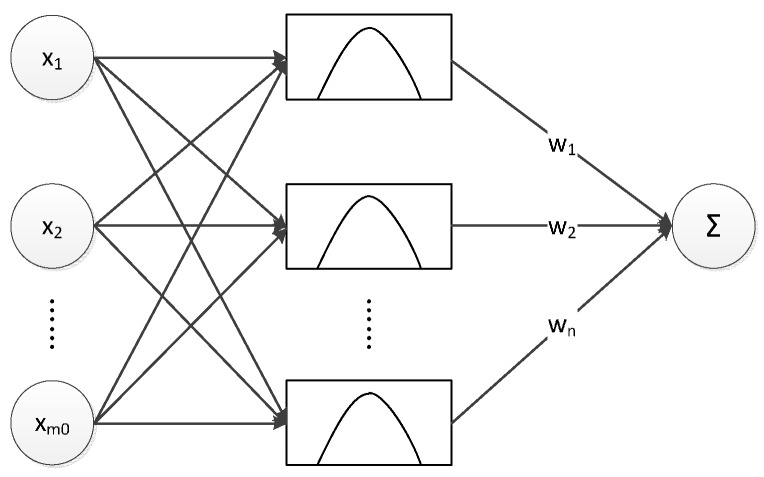
Schematic illustration of radial basis function (RBF)-artificial neural network (ANN).

In the 1980s, ANN technology became popular for dealing with practical problems. As it is inspired by biological neural networks, it shares some features with the human brain, particularly learning by example. Radial basis F = function (RBF) network is a type of ANN which uses radial basis function as the activation function. Because of the RBF network’s ability to produce optimal approximate solutions and local learning, it is used in function approximation, system control, *etc*. [16]. The RBF ANN structure is displayed in Figure 1, where {*x*_1_, *x*_2_*…x_m_*_0_} is the input vector; *m*_0_ is the dimension of the vector; *w*_1_, *w*_2_, …, *w_n_* are the connection weights between the middle layer and the output layer; and *N* is the number of the radial basis functions in the middle layer. As shown in Figure 1, the RBF ANN consists of three layers: the input layer, the middle layer and the output layer. The input layer is composed of m_0_ source points which connect the ANN to the external environment.

The second layer is the only hidden layer in the RBF network. Its function is to transform the input space into the hidden space nonlinearly. The hidden layer consists of *N* cells that can be defined mathematically by the radial function shown in Equation (5). The RBF network is good at local approximation because the radial function in the hidden layer responds to theinput partially.

(5) $$\varphi_{j}\left(x\right)=\varphi \left(\|x-x_{j}\|\right),\;j=1,2,\cdot \cdot \cdot \cdot \cdot \cdot ,\;N$$

where *x_j_* means the center of the radial function defined by the *j^th^* source point; and *x* is the signal which directly acts on the input layer. Additionally, the Gaussian function is the most widely used radial function, and the cells in the hidden layer can be defined as in Equation (6) shown below.

(6) $$\varphi_{j}\left(x\right)=\varphi \left(x-x_{j}\right)=\exp\left(-\frac{1}{2\sigma_{j}{}^{2}}\|x-x_{j}{\|}^{2}\right)\;j=1,2,\cdot \cdot \cdot \cdot \cdot \cdot ,\;N$$

where *σ_j_* is the width of the *j^th^* *x_j_*-centered Gaussian function, *x_j_* is the center of the *j^th^* basis function, *‖x* − *x_j_‖* is the vector norm of *x* − *x_j_* which means the distance between *x* and *x_j_*. Finally, the nodal points in the output layer will generate the output data.

RBF ANN is one type of feedforward static neural network. The feedforward network is the simplest network as the information can only move in one direction. The original feedforward network is a single perceptron layer network based on other networks consisting of multiple layers of computational units such as the RBF network. The RBF network is able to fit a continuous nonlinear process in a satisfied precision by automatically adjusting the weight of the functions in the hidden layer. Some studies indicate that the ANN has advantages in dealing with nonlinear problems. Ghandehari *et al*. [17] discuss the advantages of RBF over the back propagation (BP) network, which is the most widely used and popular feedforward network. Fathi and Aghakouchak [18], as well as Abdalla and Hawileh [19] applied the RBF network to fatigue crack problems successfully. In this paper, the RBF network was chosen due to its multiple advantages; in view of the RBF network’s capacity, it is suitable for establishing the function between fatigue crack growth and the driving parameters.

### 2.2. The Establishment and Training of the Artificial Neural Network (ANN)

**Figure 2:**
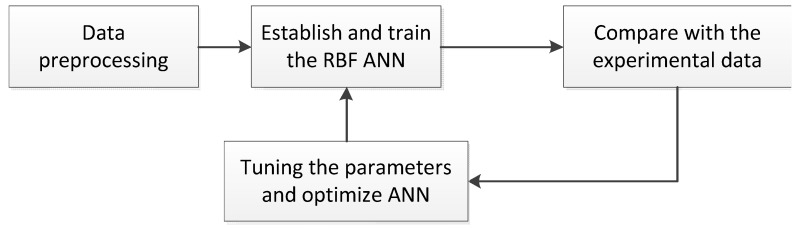
Procedure of developing a well-trained ANN.

In this section, the MATLAB (©1984–2011 MathWorks. All rights reserved, MathWorks, Natick, MA, USA) software is used to establish and train the RBF-ANN as shown in Figure 2. First of all, a multi-input single-output RBF-ANN is established by analyzing the physical process of fatigue crack growth. The raw experimental data then need to be preprocessed before training the ANN. The data preprocessing includes two steps: the first step is to take the logarithm of Δ*K* and *da*/*dN* to reduce the influence from the order of the magnitude; the second step is to normalize the data from the first step. After preprocessing, the experimental data have been transformed into a number of vectors, which are used to train the ANN. ANN can be trained automatically by using the MATLAB toolbox. During the training, some parameters can be tuned for optimization, including: the mean square error (MSE) goal, expansion speed of RBF, maximum number of neurons, *etc*. For example, the MSE goal controls the fitting accuracy. By comparing the output with the testing data and balancing the accuracy and efficiency, the optimal tuning parameters can be determined.

#### 2.2.1. The Constant Amplitude Loading

**Figure 3:**
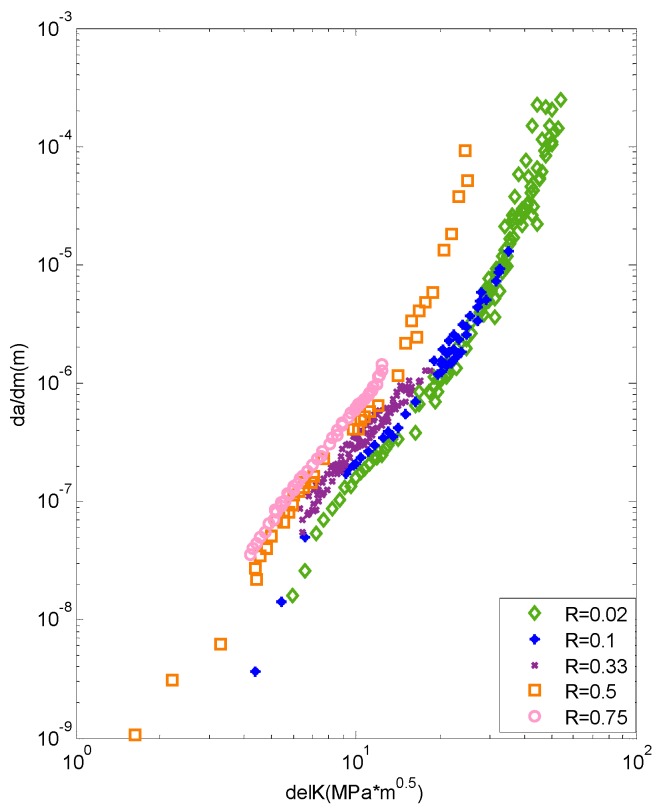
The experimental data of Al7075-T6.

The experimental data [20] are plotted in Figure 3. The *x*-axis is the SIF range; the *y*-axis is the crack growth rate; and the different kinds of dots represent the testing data with different stress ratios. It can be seen that the testing data do not follow a perfect linear tendency.

With the experimental data in Figure 3, the ANN can be trained following the procedure in Figure 2. For the constant amplitude loading, the plasticity, on behalf of the historical load, is proportional to the current loading. The SIF and stress ratio are therefore chosen to be the inputs, and the crack growth rate is the output. The training vectors are preprocessed to make them suitable for the ANN. During training, the ANN can learn deeply from the limited data and establish the continuous function between the inputs and the output.

**Figure 4:**
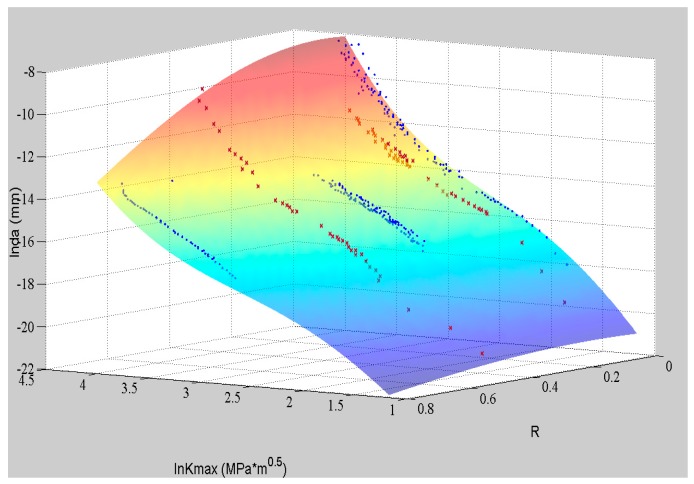
The experimental data of Al7075-T6 and the corresponding ANN.

The fitting surface by well-trained ANN and the testing data are shown in Figure 4. The blue dots represent the training data, and the red crosses are the data for validation. It can be observed that the fitting surface can match all the experimental data well, even though they are not perfectly log-linear. The nonlinearity of the data can be studied by the ANN so that its prediction has a higher accuracy than the tradition log-linear formulas. Moreover, the ANN can offer a continuous predicting surface in the domain of definition based on the limited and discrete training data. This example shows ANN’s advantage in fitting and extrapolating the crack growth rate under constant amplitude loading with different stress ratios.

#### 2.2.2. Single Overload

For the variable amplitude loading, the load interaction effects cannot be ignored, because the influence of historical loading sequence is dependent on the current load cycle. Single overload, as the simplest and most typical variable amplitude loading, is investigated in this paper to demonstrate the loading interaction effect.

As is well known, an applied overload can lead to fatigue crack growth retardation or even crack arrest. This phenomenon is caused by the loading interaction effect, and its existence obviously stimulates nonlinear damage accumulation. Wheeler [5], De Koning [6] and many other researchers [21,22,23,24,25,26,27] have introduced additional parameters to describe the influence of the historical loading sequence. Wheeler [5] models the retardation by correlating the plastic zone size ahead of the crack tip with the crack growth rate. Topper and Yu [28] use the plasticity-induced crack closure to explain this phenomenon. Above all, the plasticity ahead of the crack tip is a reasonable parameter to account for the loading interaction effect. In this paper, a concept “equivalent stress intensity factor”, which is derived from the equivalent plastic zone, is employed as input data to handle the nonlinear damage accumulation. The details are discussed in Section 3.2.1.

### 2.3. A Fatigue Life Prediction Method

There are three steps to calculating the fatigue crack length. First the crack increment within one load cycle is computed; the the crack length, the geometric factor, and the SIF are subsequently updated, thereby preparing the inputs for the next cycle. In repeating this process, the fatigue crack propagation is simulated cycle by cycle. The framework is shown in Equations (7) and (8).

(7) $$\{\begin{array}{c} a_{I}=a_{0}+\sum_{j=1}^{I}da_{j}=a_{0}+\sum_{j=1}^{I}g\left(\Delta K_{j},R,\cdots \right)\\ \frac{da}{dI}=g\left(\Delta K_{j},R,\cdots \right)\\ \Delta K_{j}=\Delta \sigma \sqrt{\pi a_{j}}\times q\\ q=Q\left(\frac{a_{j}}{w}\right) \end{array}$$

where *a_I_* is crack length in the *I^th^* cycle; *a*_0_ is the initial crack length; g(Δ*K_j_*, *R*, …) denotes the general relationship between the crack growth rate and applied load; *da_j_* is the increment during the *j^th^* cycle; Δ*σ* is the stress amplitude; *Q* is the geometric factor while *w* is the width of the specimen. Furthermore, once the failure criterion or the critical crack length is provided, the fatigue life can be determined.

**Figure 5:**
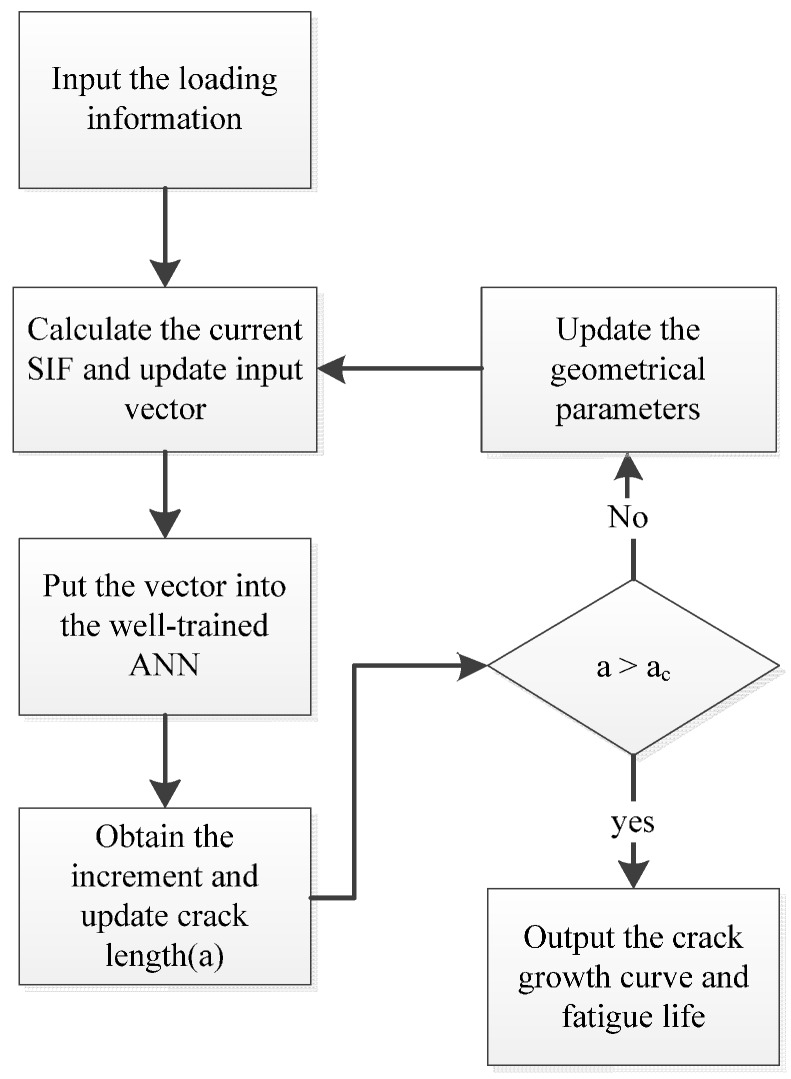
The flow chart of the ANN-based algorithm.

In this study, the ANN is used to quantify the relationship between the loading and the crack increment per cycle instead of the traditional equation. Equation (7) can therefore be transformed into Equation (8).

(8) $$a_{I}=a_{0}+\overset{I}{\underset{j=1}{\sum }}f_{ANN}\left(\Delta K_{j},R,\cdot \cdot \cdot \right)$$

where *f_ANN_* (Δ*K*, *R*, …) represents a general ANN function describing the relationship between driving parameters and crack growth rate. Generally, the driving parameters would include SIF range (Δ*K*), stress ratios, plastic zone, *etc*. Additionally, an ANN-based framework for fatigue crack growth calculation can be established. The flow chart is shown in Figure 5.

## 3. Validation and Comparison

### 3.1. Validation and Comparison of the Constant Loading with Different Stress Ratios

#### 3.1.1. ANN Training

As the ANN can quantify the relationship from experimental data, it is significant to select the suitable data for the training. In this section, the testing data [20] of 7075-T6 aluminum alloy is used to train the ANN globally. The information of the experiment is listed in Table 1.

With these experimental data the relationship between the loading and fatigue crack growth rate can be fitted by the ANN.

**Figure 6:**
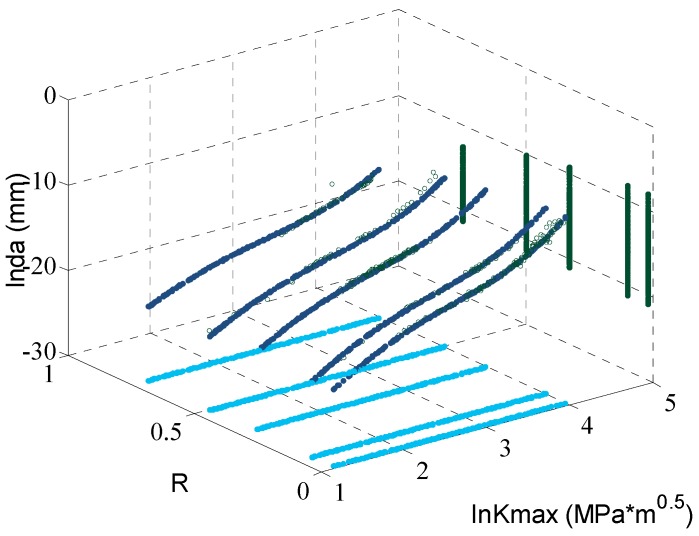
The fitting curves of ANN trained with all data.

At first, the ANN is trained by all five sets of the experimental data with different stress ratios. The fitting curves are plotted with the original data in Figure 6. In this figure, the *x*-axis is the stress ratios (from 0 to 1); the *y*-axis is the SIF in logarithmic coordinate; and the *z*-axis is the crack growth rate in logarithmic coordinate. The blue cycles represent the experimental data; and the dark blue lines are the ANN prediction. It is observed clearly that the curves fit the experimental data well. Additionally, the projections of the fitting curves are also provided.

**Figure 7:**
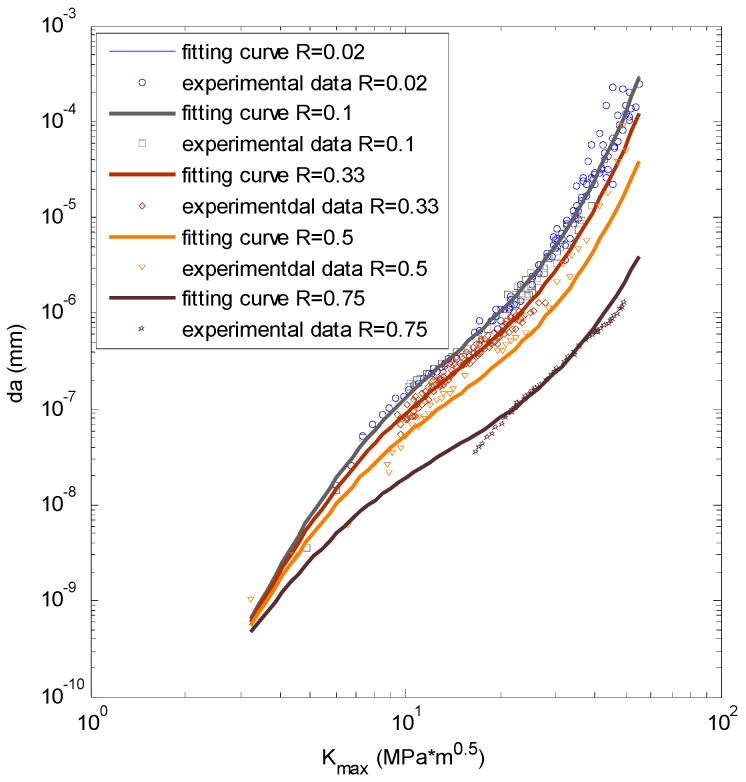
The fitting by ANN *vs.* the testing data for Al7075-T6.

To observe the fitting accuracy clearly, Figure 7 displays the prediction and the experimental data in a 2D plot. From the picture it can be seen that the nonlinear fitting curves by ANN can fit the experimental data well.

**Figure 8:**
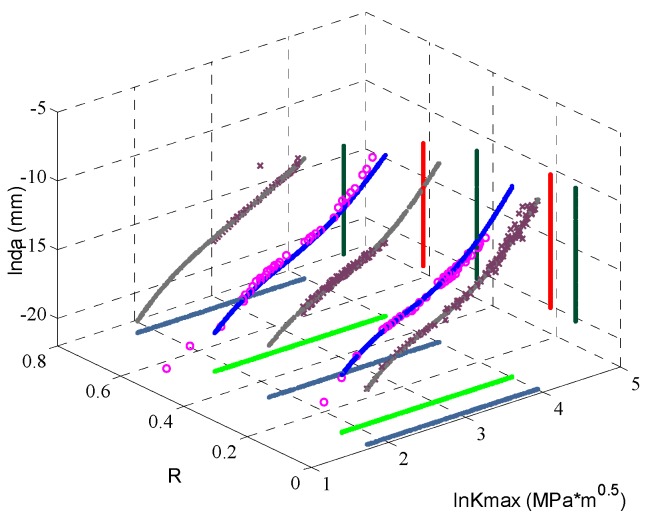
The fitting curves of ANN trained with part of data.

In this part the impact from data size on the fitting performance is investigated. This time the training vectors only include thre sets of experimental data with stress ratios 0.02, 0.33 and 0.75; the other experimental data are used for validation. The ANN prediction and the experimental data are shown in Figure 8. In this picture only the purple crosses are the experimental data used to train the ANN. It is obvious that the fitting accuracy is still satisfactory compared with Figure 8.

**Figure 9:**
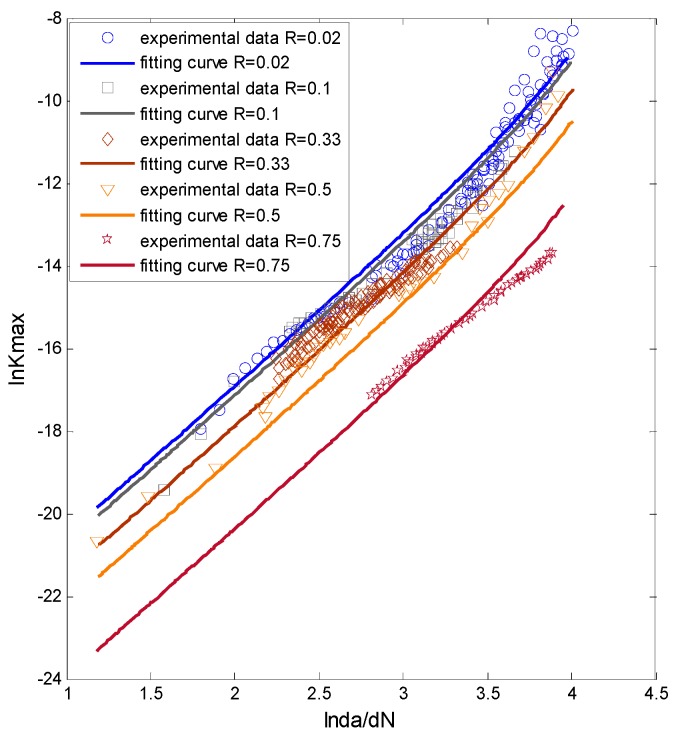
The fitting by Forman’s model *vs.* the testing data for Al7075-T6.

Forman’s equation is also utilized to calculate fatigue crack growth under different stress ratios to make a comparison. Table 2 shows the calibration indices of Equation (9). The fitting parameters are calibrated with the global database. The prediction by Equation (9) is displayed in Figure 9. It can be concluded that the predictions by Forman’s equation are linear in log-log coordinate while the ANN prediction curves are nonlinear. where *r* is the coefficient of association; *RMSE* is the root-mean-square error; *SSE* is the sum of squares for error and *DC* is the determination coefficient.

(9) $$\frac{da}{dN}=\frac{2.9838\times 10^{-9}\times \Delta K^{3.5241}}{\left(1-R\right)K_{c}-\Delta K}$$

With the good performance of ANN under the discrete *R* values, it is reasonable that the ANN can deliver a good fitting surface within the continuous domain as shown in Figure 4.

**Figure 10:**
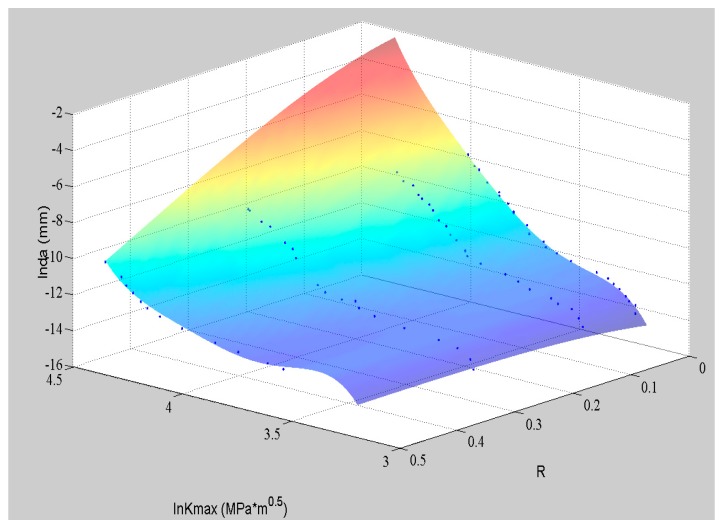
The experimental data of Al2024-T315 and the corresponding ANN.

Furthermore, additional material is utilized to test the ANN. Figure 10 shows the fitting surface by ANN and experimental data of Al2024-T315 [21]. Four sets of experimental data (stress ratios: 0, 0.1, 0.33 and 0.5) are all used to train the ANN globally.

**Figure 11:**
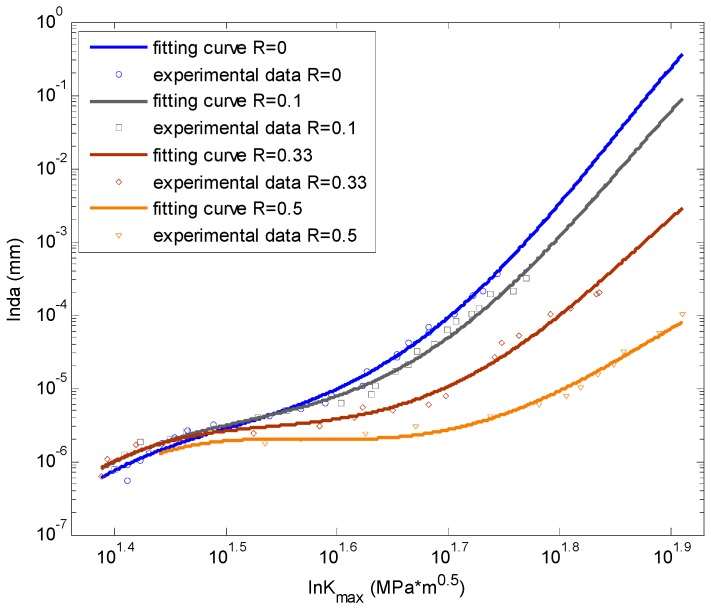
The fitting by ANN *vs.* the testing data for Al2024-T315.

To observe the fitting accuracy clearly, Figure 11 shows the prediction and the experimental data in a 2D plot. From the figure it can be seen that the nonlinear fitting curves by ANN can fit the experimental data well.

#### 3.1.2. Crack Growth Calculation under Constant Amplitude Loading

**Figure 12:**
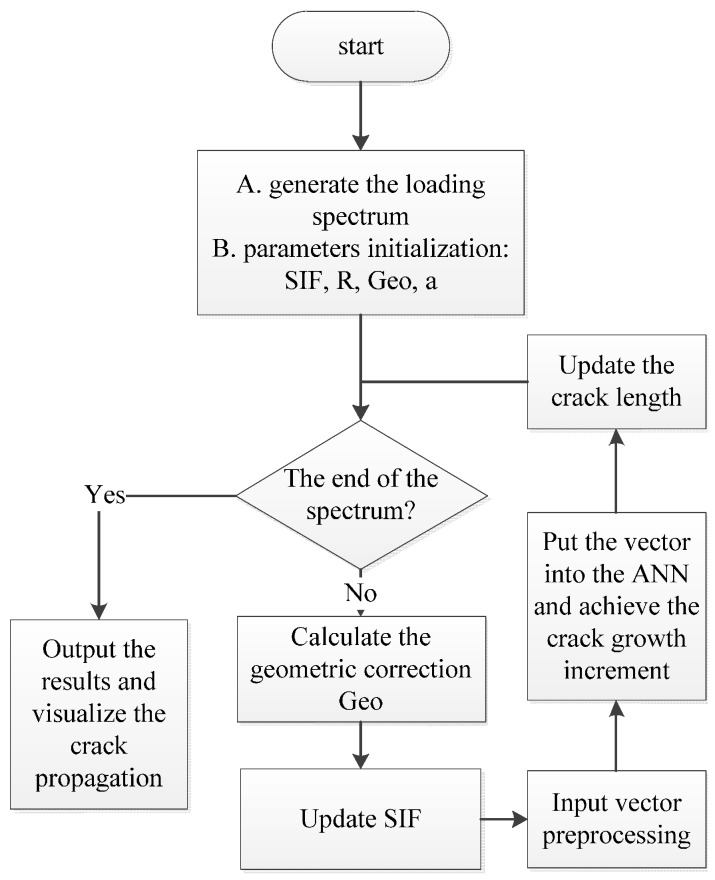
Flow chart of programing the predicting algorithm.

With the well-trained ANN, the algorithm for the crack propagation is programmed by MATLAB and the flow chart is shown in Figure 12. At first, a loading spectrum is generated and the parameters are initialized. The input vector is then prepared following the same procedure in Section 2.2. After that, this vector is entered into the well-trained ANN and the crack increment is worked out. With the crack increment in the current cycle, the crack length gets updated for the next iteration. When the whole loop is repeated until the last cycle, the simulation of the fatigue crack propagation is accomplished.

To validate the ANN-centered algorithm, the experimental data of Al7075-T6 are used [20]. The ANN has been trained with the *da*/*dN*-Δ*K* data as shown in Figure 4. Some additional data (a-N curves) are then utilized to compare with the model prediction. In Table 3, the testing information of these a-N curves are listed. Moreover, Forman’s equation also serves as a comparison.

**Figure 13:**
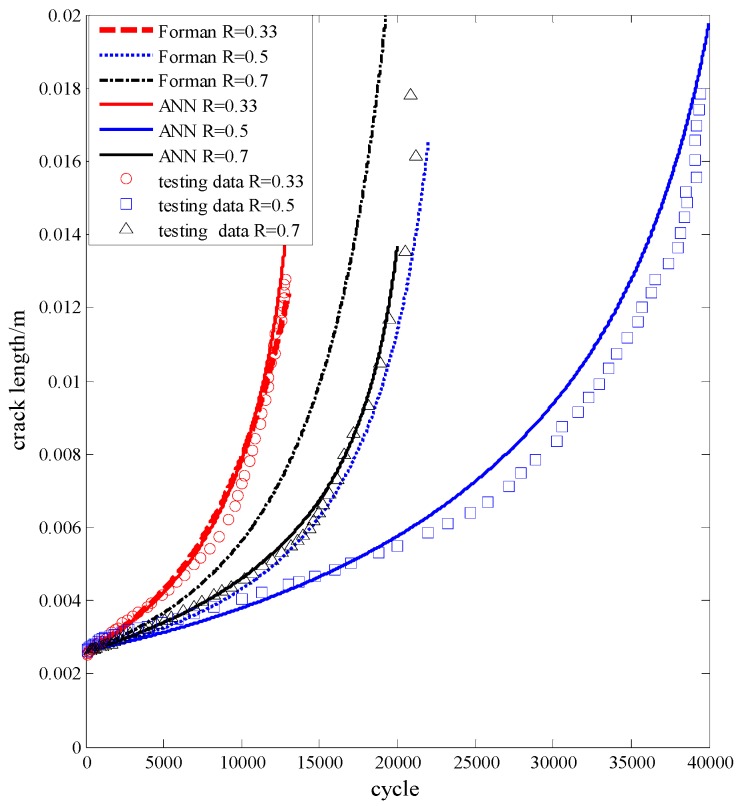
The predictions *vs.* the experimental data for Al7075-T6 aluminum alloy.

In Figure 13, the *x*-axis is the cycle number, and the *y*-axis is the length of the crack. The experimental data and the predictions by the two different models are all visualized in different lines. It is obvious that the performance of ANN is better than Forman’s model.

Once the failure criterion is given, the corresponding fatigue life can be determined. Assuming that the critical crack length is 0.008 m, 0.01 m, and 0.012 m, the corresponding errors of the two models are shown in Table 4. It is evident that the accuracy and stability of ANN is much better than Forman’s equation.

**Figure 14:**
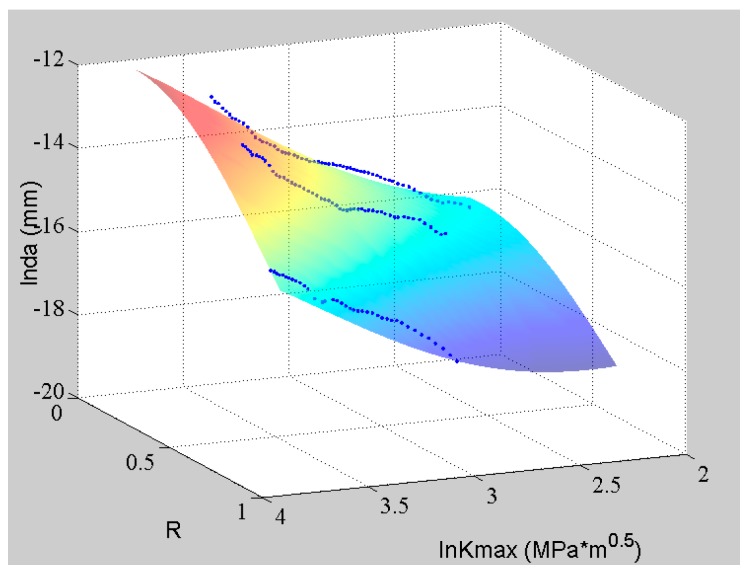
The experimental data of D16 aluminum and the corresponding ANN.

**Figure 15:**
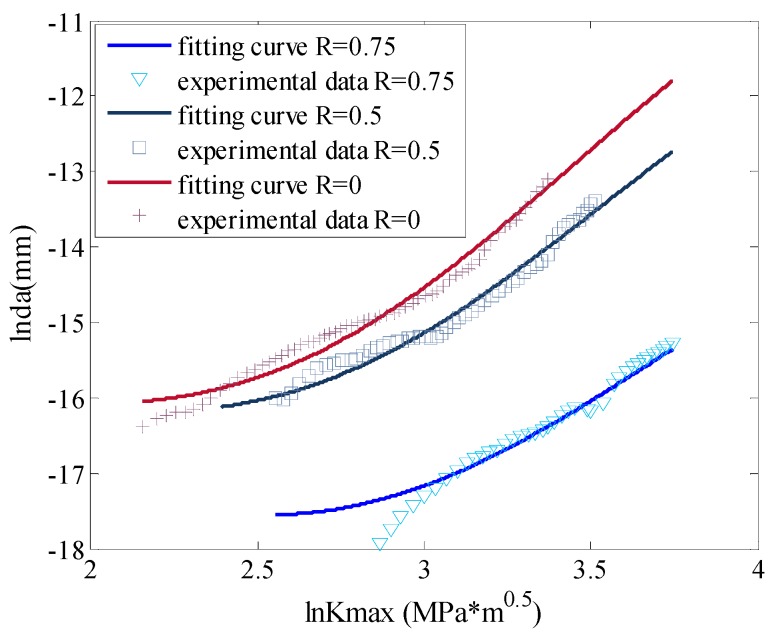
The fitting by ANN *vs.* the testing data for D16 aluminum alloy.

Furthermore, additional testing data in D16 aluminum alloy are used for model validation. The information of the experiment is listed in Table 5 [29]. Similarly, the ANN is trained with the crack growth rate data (*da*/*dN*-Δ*K*) under three stress ratios. The 3D fitting surface and the 2D projections of the ANN are shown in Figure 14 and Figure 15, respectively.

**Figure 16:**
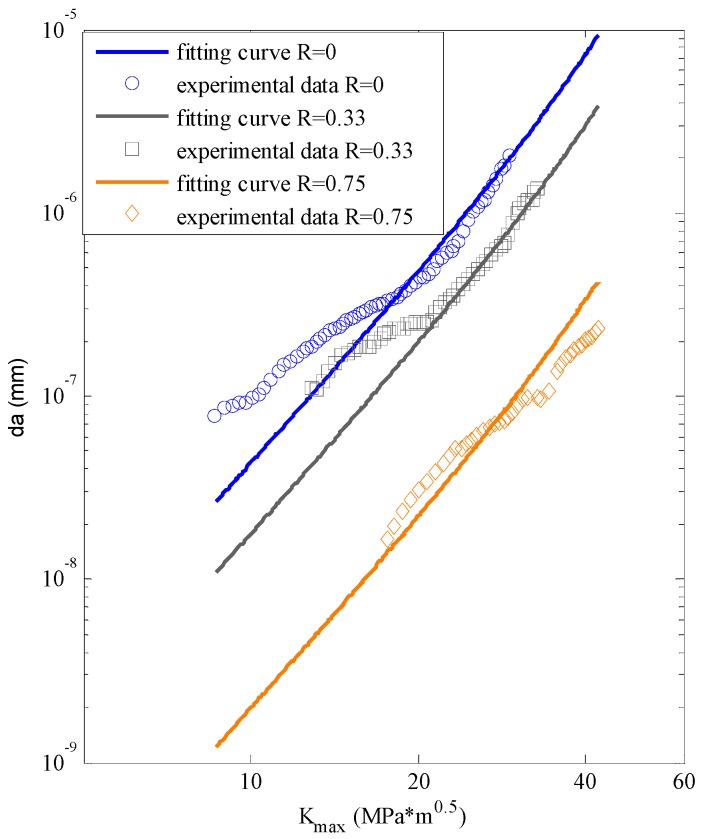
The fitting by Forman’s model *vs.* the testing data for D16 aluminum alloy.

Forman’s model is still employed for comparison. The calibrated equation is Equation (10) and the fitting indices are listed in Table 6. Figure 16 shows the fitting lines of Forman’s equation.

(10) $$\frac{da}{dN}=\frac{1.5903\times 10^{-9}\times \Delta K^{3.2215}}{\left(1-R\right)K_{c}-\Delta K}$$

**Figure 17:**
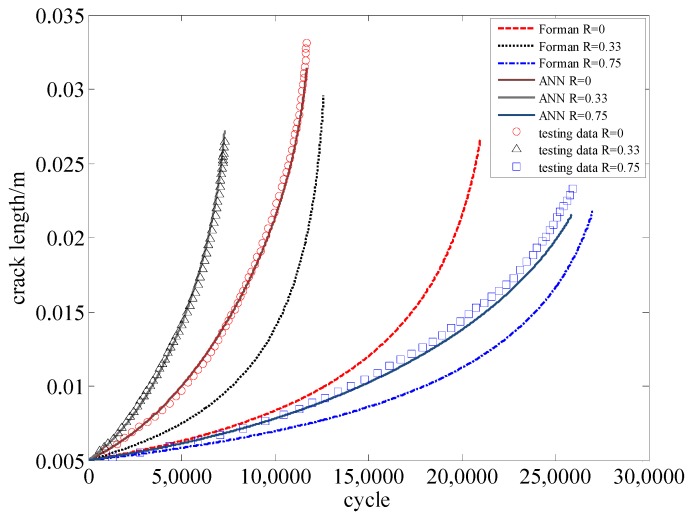
The predictions *vs.* the experimental data for D16 aluminum alloy.

Similarly, the experimental data of D16 are used to validate the algorithm. Some additional data (a-N curves) are utilized to compare with the model prediction. In Table 7 the testing information of these a-N curves are listed. In Figure 17 the experimental data and the predictions by the two different models are plotted together. It is clear that the results of the proposed model match the testing data better than those by Forman’s model.

Assuming that the critical crack length is 0.015 m, 0.018 m, and 0.020 m, the relative errors of the two models are compared in Table 8. It is clear that the proposed model has very high accuracy and stability.

### 3.2. Validation and Comparison of the Constant Loading with a Few Overloads

#### 3.2.1. Equivalent Stress Intensity Factor

**Figure 18:**
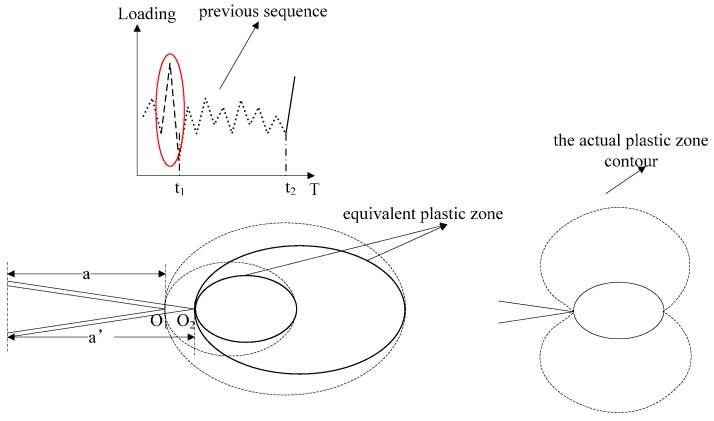
Schematic illustration of the equivalent plastic zone concept.

It is indicated that the plasticity ahead of the crack tip affects fatigue crack growth behavior. The retardation effects due to overload can be correlated with the plastic deformation. The plastic state caused by the previous loads is traced. Subsequently, the equivalent stress intensity factor is calculated, which is based on the equivalent plastic zone concept. The general expression of the equivalent plastic zone can be written as:

(11) $$a_{0}+\overset{i}{\underset{j=1}{\sum }}da_{j}+D_{eq,i}=\text{max}\left\{a_{0}+\overset{i}{\underset{j=1}{\sum }}da_{j}+d_{i},a_{0}+\overset{i-1}{\underset{j=1}{\sum }}da_{j}+D_{eq,i-1}\right\}$$

where *D_eq.i_* means the size of equivalent plastic zone in the *i^th^* cycle; *a*_0_ means the initial crack length; da means the crack increment; d_i_ means the current plastic zone size in the *i^th^* cycle; $a_{0}+\sum_{j=1}^{i}da_{j}$ means the crack length in the *i^th^* cycle; *i* means the current cycle number. A schematic sketch is given to illustrate the equivalent plastic zone concept. The loading sequential process and the corresponding plastic state variation are shown in Figure 18. The dashed zigzag lines represent the loading history. The large plastic zones have been formed at “t_1_,” and the crack tip is “O_1_” at that moment. The monotonic and reverse plastic zones can be expressed as Equation (12) [30]:

(12) $$\{\begin{array}{c} d_{m}=\frac{\pi }{8}{\left(\frac{K_{max}}{\sigma_{y}}\right)}^{2}\\ d_{r}=\frac{\pi }{8}{\left(\frac{K_{max}-K_{op}}{2\sigma_{y}}\right)}^{2} \end{array}$$

where *d_m_* is the monotonic plastic zone size; and *d_r_* is the reverse plastic zone size. The current load is applied at “t_2_” and the new crack tip is “O_2_”. The large forward and reverse plastic zones, which are the dotted ellipses, form during the largest load cycle in the previous loading history. Before “t_2_”, the following plastic zones do not reach their boundaries respectively even though the crack grows. The solid ellipses represent the equivalent plastic zones ahead of the crack tip O_2_. In addition, the actual contour of the plastic zone is butterfly-shaped instead of round; theoretically, however, their diameters along the crack direction are identical, as shown in Figure 18. In the current investigation, the equivalent plastic zone is in directly proportional to the circular diametric distance and the proportionality coefficient is equal to or slightly greater than 1. Equation (12) can be rewritten as:

(13) $$\{\begin{array}{c} a_{0}+\sum_{j=1}^{i}da_{j}+D_{m,eq,i}=\max\{a_{0}+\sum_{j=1}^{i}da_{j}+d_{m,i},a_{0}+\sum_{j=1}^{i-1}da_{j}+D_{m,eq,i-1}\\ d_{m,i}=\Psi *\frac{\pi }{8}{\left(\frac{K_{max,i}}{\sigma_{y}}\right)}^{2}\\ a_{0}+\sum_{j=1}^{i}da_{j}+D_{r,eq,i}=max\{a_{0}+\sum_{j=1}^{i}da_{j}+d_{r,i},a_{0}+\sum_{j=1}^{i-1}da_{j}+D_{r,eq,i-1}\\ d_{r,i}=\Psi *\frac{\pi }{8}{\left(\frac{K_{max,i}-K_{op,i}}{2\sigma_{y}}\right)}^{2} \end{array}$$

where *D_m_*_,*eq,i*_ and *D_r_*_,*eq,i*_ are the equivalent monotonic and reverse plastic zone in *i^th^* cycle respectively; *Ψ* is the geometry modification factor of plastic zone.

The equivalent stress intensity factors K_E_ can be calculated by solving the following equation:

(14) $$K_{E}=\sigma_{S}\sqrt{\pi D_{m,eq,i}}$$

where *D_m,eq,i_* means the plastic zone in this cycle and *σ_s_* means the yield limit.

#### 3.2.2. Single Overload

**Figure 19:**
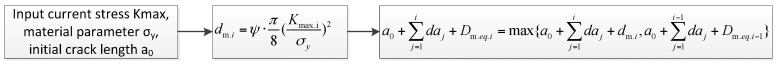
The calculation of the equivalent plastic zone.

Unlike the constant amplitude loading case, the algorithm for the single overload needs an additional parameter called equivalent stress intensity factor to account for the nonlinear loading interaction effect. To obtain this parameter, the equivalent plastic zone has to be calculated. Figure 19 shows the procedure to calculate the equivalent plastic zone, where *D_m,eq,i_* means the plastic zone which characterizes the influence of the history load. Once *D_m,eq,i_* is estimated following the flow chart, the equivalent SIF can be calculated by using Equation (14). Then the ANN can get trained by using the training data vectors, in which the equivalent SIF, SIF and stress ratios are inputs and the corresponding crack growth rate is the output. Additionally, all the training data have to be preprocessed following the procedure in Section 2.2. At last the fatigue crack growth with retardation can be estimated.

The experimental data in D16 aluminum alloy [29] are employed to validate the model. The basic information about the experiment can be seen in Table 5. *da*/*dN*-Δ*K* curve serves as the training vector, and the a-N curve is used to validate the whole prediction algorithm.

**Figure 20:**
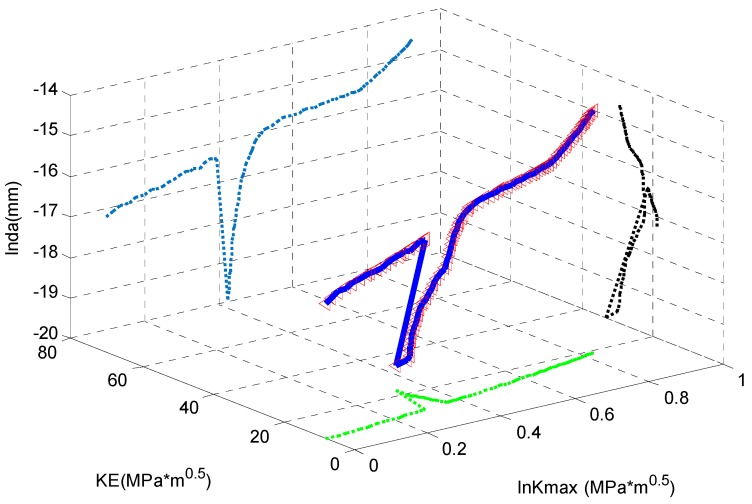
The fitting *vs.* the experimental data for D16 aluminum alloy.

As shown in Figure 20, the *x*-axis is the equivalent SIF; the *y*-axis is the *K*_max_; and the *z*-axis is the crack growth rate. The red small triangles are the experimental data; the blue curve represents the well-trained ANN; and three broken curves are its projections. It is obvious that the curve by ANN can fit the highly nonlinear tendency of the experimental data perfectly.

**Figure 21:**
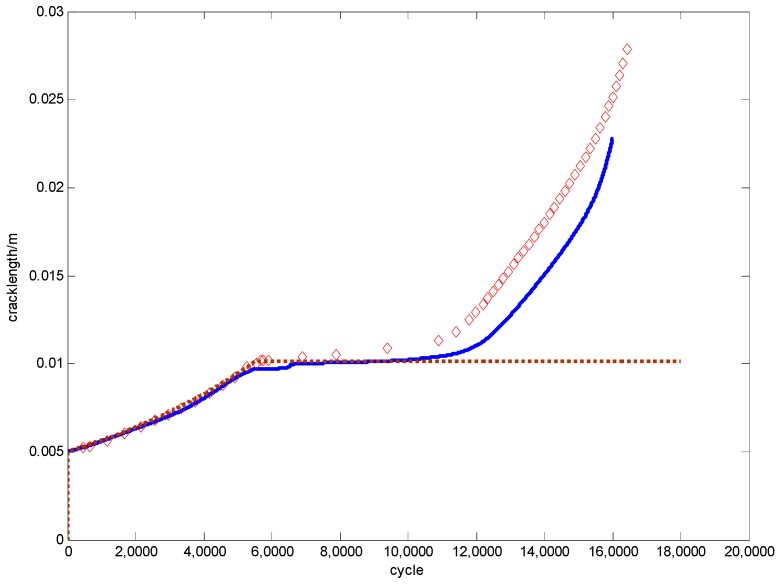
The predictions *vs.* the experimental data for D16 aluminum alloy.

Fatigue crack growth with the overload effect is thus simulated as shown in Figure 21. The extra experimental information for a-N curve is listed in Table 9, where the S_ol_ is the overload stress level. The prediction by Wheeler’s model is also given for comparison.

The figure shows that there is an overload applied when the crack length reaches 0.01 m. After that, a conspicuous retardation phenomenon can be seen. The slope of the curve decreases dramatically until the crack grows out of the retardation effect area after another 60,000 cycles. The prediction by the ANN-based approach has a very good agreement with the testing data in this figure. However, the curve by Wheeler’s model stops growing after the overload is applied. Ribeiro *et al.* [31] indicate that Wheeler’s model has some difficulties in crack growth calculation when the overload is larger than twice that of the *σ*_max_. However, the approach proposed in this paper does not have this kind of problem which makes it more generally applicable.

#### 3.2.3. Multiply Overloads

Other testing data for different materials are employed here for the model validation [32]. The information of this experiment is listed in Table 10.

**Figure 22:**
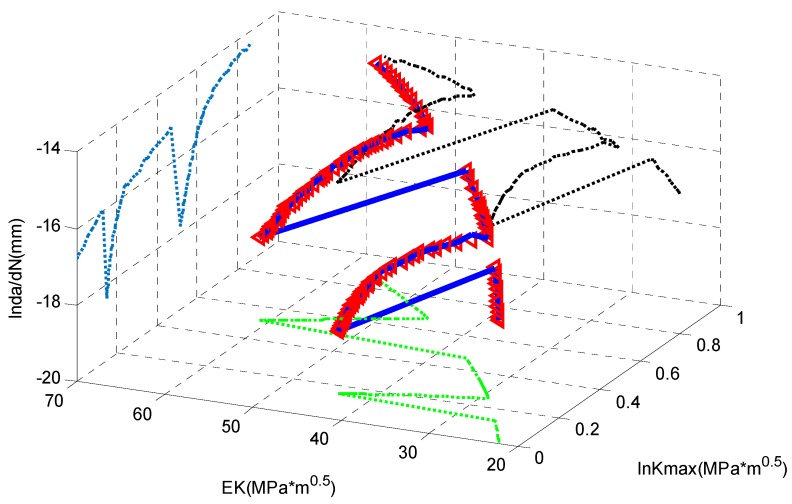
The fitting *vs.* the experimental data for 350 WT steel.

Similarly, the ANN is trained by the experimental data and the result is shown in Figure 22. The small red tangles are the experimental data; the blue curve is the fitting by the ANN; and the three broken curves are its projections. It is seen that the ANN delivers a good fitting.

**Figure 23:**
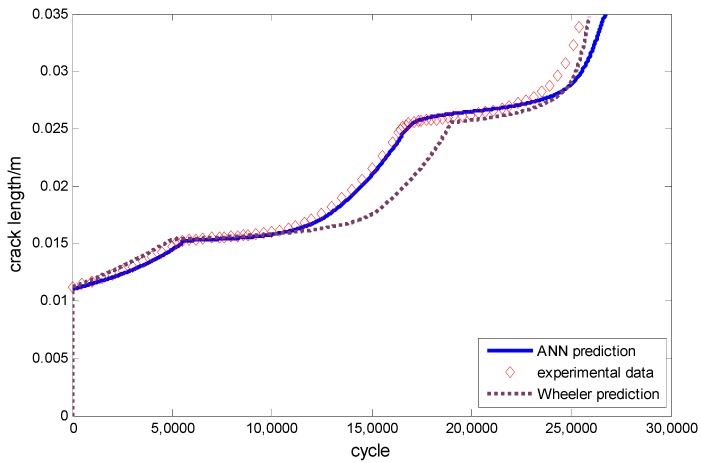
The predictions *vs.* the experimental data for 350 WT steel.

The predictions made by the ANN-based approach and Wheeler’s model are visualized in Figure 23 with the experimental data as well. Both the methods give good agreements with the testing data. The proposed model performs slightly better around the 150,000th cycle.

From the validations above, it can be concluded that the proposed method can deal with the nonlinear and multivariable fatigue damage accumulation process successfully.

## 4. Conclusions and Future Work

In this paper, a novel method to predict the fatigue crack growth based on a radial basis function (RBF)-artificial neural network (ANN) is developed. The ANN-centered algorithm is also validated by comparison with the experimental data under the constant and variable amplitude loading of different materials. Forman’s and Wheeler’s models are also employed for comparisons. It is clear that the proposed model has very high accurate and stable performance in all the examples.

All the validations above prove the advantages of the ANN-based algorithm in nonlinear fatigue crack growth problems. This method still has some limitations that need further investigation. One major issue is that the size of the training data has a significant impact on the prediction accuracy. The other is that the method may be time consuming and computationally expensive due to its cycle-by-cycle nature.

## Figures and Tables

**Table 1:** The experimental information of Al7075-T6.

Material	Al7075-T6
Specimen type	Middle cracked tension specimen
Specimen length	889 mm
Specimen width	305 mm
Specimen thickness	2.28 mm
Initial crack length	2.5 mm
Loading type	Tension-tension, constant amplitude

**Table 2:** The fitting indices of Al7075-T6.

The Fitting Indexes	Number
*r*	0.796
*Chi-Square*	0.000513
*RMSE*	1.47 × 10^−5^
*SSE*	7.52 × 10^−8^
*DC*	0.796

**Table 3:** Loading information for the a-N curves.

*R*	*σ*_min_	*σ*_max_
0.33	51.2 MPa	155 MPa
0.5	69 MPa	138 MPa
0.7	168.7 MPa	241 MPa

**Table 4:** The errors of the results by two models for Al7075-T6.

ac	*R*	ANN Algorithm	Forman Algorithm
0.008	0.33	−4.72%	−20.85%
	0.5	−2.18%	4.73%
	0.75	−1.20%	−34.34%
0.01	0.33	−2.59%	−21.38%
	0.5	−6.06%	−8.48%
	0.75	−1.61%	−37.10%
0.012	0.33	−3.97%	−23.10%
	0.5	−10.92%	−4.76%
	0.75	−2.03%	−38.07%

**Table 5:** The experimental information of D16 aluminum alloy.

Specimen Material	D16 Aluminum Alloy
Crack type	Middle cracked tension specimen
Specimen length	500 mm
Specimen width	100 mm
Specimen thickness	0.04 mm
Initial crack length	10 mm
Loading type	Tension-tension, constant amplitude

**Table 6:** The fitting indices of D16.

Index	Number
*r*	0.984
*Chi-Square*	1.93 × 10^−6^
*RMSE*	7.80 × 10^−8^
*SSE*	9.24 × 10^−13^
*DC*	0.964

**Table 7:** Loading information for the a-N curves.

*R*	*σ*_min_	*σ*_max_
0.75	105 MPa	140 MPa
0.33	96 MPa	32 MPa
0	64 MPa	0 MPa

**Table 8:** The errors of the results by two models.

ac	*R*	ANN Algorithm	Forman Algorithm
0.015	0	−1.40%	120.95%
	0.33	−1.32%	96.60%
	0.75	2.90%	15.46%
0.018	0	−1.13%	111.78%
	0.33	−3.26%	82.71%
	0.75	2.16%	10.82%
0.02	0	1.05%	105.70%
	0.33	−1.69%	80.00%
	0.75	2.46%	8.61%

**Table 9:** Applied loading for D16 specimen.

Type of Loading	S_min_	S_max_	ΔS	*R*	S_ol_
CA + single overload	0 MPa	64 MPa	64 MPa	0	128 MPa

**Table 10:** The experimental information of 350 WT steel.

Specimen Material	350 WT Steel
**Crack Type**	**Center Cracked Tension Specimen**
Specimen length	300 mm
Specimen width	100 mm
Specimen thickness	5 mm
Initial crack length	20 mm
**Type of loading**	**Tension-tension, Constant amplitude with overload**
S_min_	11.4 MPa
S_max_	114 MPa
S_ol_	190.95 MPa
